# Acceptability of the Expert Standard for Oral Health Care in Older Adult Patients Among Nursing Staff in German Hospitals and Care Facilities: Protocol for a Cross-Sectional Study

**DOI:** 10.2196/72528

**Published:** 2025-06-03

**Authors:** Damian Pazdziernik, Harald Stummer

**Affiliations:** 1 Department of Public Health, Medical Decision Making and Health Technology Assessment Institute for Management and Economics in Healthcare UMIT TIROL – Private University for Health Sciences and Health Technology Hall in Tirol Austria; 2 Institute for Health Management IMC University of Applied Sciences Krems Krems Austria

**Keywords:** oral health, nursing standards, acceptability, competence, missed nursing care, health intervention feasibility, long-term care, interprofessional collaboration, implementation science

## Abstract

**Background:**

The aging population and increasing prevalence of natural teeth among older adults have escalated the demand for oral health care, especially in nursing settings. Impaired oral health in older individuals is closely linked to systemic conditions such as diabetes and cardiovascular diseases. The expert standard “promoting oral health in nursing” was developed in Germany to enhance the quality of oral care and address future challenges in geriatric nursing. It comprises a series of recommended interventions targeting oral health promotion in nursing care. However, significant barriers, including high patient-to-nurse ratios and staff shortages, often result in missed or rationed nursing care, limiting the feasibility and implementation of such interventions. Evaluating the acceptability of this standard is critical to its successful integration into routine nursing practice.

**Objective:**

We aim to assess the acceptability of the expert standard among nursing staff providing care for older individuals, identify factors influencing its adoption, and examine the relationship between nursing competence, care rationed or missed (CROM), and the standard’s acceptability.

**Methods:**

This quantitative cross-sectional study will collect data from nursing staff in 25 hospitals and long-term care facilities in North Rhine-Westphalia, Germany, using standardized survey instruments. Based on the template of the generic theoretical framework of acceptability, a questionnaire to measure the acceptability of interventions across 7 domains was created. Oral health knowledge will be assessed using the Oral Health Literacy Profile and competence in mouth care using the questionnaire developed by the DNQP (Deutsches Netzwerk für Qualitätsentwicklung in der Pflege [German network for Quality Development in Nursing]). Barriers to implementation will be evaluated according to the acute care nurses’ questionnaire on oral hygiene and CROM using the oral care–related question from the Basel Extent of Rationing of Nursing Care instrument. Statistical analyses consist of first calculating the mean acceptability with a 95% CI for each recommended intervention of the expert standard. Second, repeated measures ANOVA is used to examine mean differences in acceptability between these interventions. Third, linear regression analyses are used to test the impact of nursing competence on acceptability and lastly chi-square tests of independence are used to compare CROM with already published rates in German-speaking countries.

**Results:**

The results are anticipated to provide insights into the acceptability of the expert standard and its determinants, including nursing competence and perceived barriers. Data collection will commence in June 2025 and is expected to be completed by October 2025.

**Conclusions:**

This study evaluates the acceptability of the expert standard for oral health in nursing. The findings will support evidence-based strategies to enhance feasibility, reduce CROM prevalence, and improve oral health in older adults. By focusing on acceptability as a prerequisite for implementation, this study emphasizes the need to align interventions with the realities of nursing care to achieve effective outcomes.

**International Registered Report Identifier (IRRID):**

PRR1-10.2196/72528

## Introduction

### Background

The ongoing demographic shift toward an older society presents growing challenges for health care systems [1,2]. Poor oral health in older adults is closely associated with systemic diseases, such as diabetes and cardiovascular disease, which require special attention [3,4]. Advances in dental prophylaxis have significantly reduced edentulism among older adults, leading to a new generation of toothed care recipients [1]. The combination of a growing older adult population and more natural teeth leads to increased demand for oral health interventions and associated nursing care. This progress highlights the increasing complexity and cost intensity of dental care, requiring greater attention and resources.

In response to these challenges, an interdisciplinary group of dentists and nurses in Germany established evidence-based interventions aimed at improving oral health in nursing care [5,6]. The resulting consensus-based expert standard, “promoting oral health in nursing,” aims to improve the quality of oral care and overall health while addressing the future challenges of nursing care. There is evidence that the use of care standards can lead to improved quality in the care of older people [7]. However, significant barriers arise from the workload of nursing staff, making the effective execution of additional measures challenging [8-12].

Current studies highlight the strain on nursing personnel caused by high patient-to-nurse ratios. Time constraints and staff shortages often lead to care rationed or missed (CROM) [8-12].

For example, in cross-sectional studies, 31% of nurses in Austrian hospitals [13], 35% in German hospitals [12], and 22% in Swiss long-term care facilities [11] reported that they had omitted oral care within the last 14 or 7 days, respectively, at least sometimes or frequently, according to a Likert-type rating scale. According to Schubert et al [10], there is no universal intervention that can reduce the CROM for all care procedures. They have found evidence that interventions can help to reduce the CROM in selected measures only [10]. These findings underscore the urgent need to consider CROM phenomena when establishing new standards.

Ensuring the feasibility of interventions under current working conditions is critical, as even the best-designed standards are ineffective if they are not adopted or executed. According to Proctor et al [14] and Skivington et al [15], acceptability is a key prerequisite for feasibility and should, as recommended by the UK Medical Research Council (MRC), be assessed prospectively. Mirbeth [16] found initial evidence that acceptance is crucial for sustaining expert standards. Acceptability of future interventions and also acceptance of interventions that have already been implemented determine whether nursing staff will adopt and apply an intervention.

Assessing the acceptance of the expert standard is therefore of central importance, particularly in the context of overburdened nursing staff [17,18].

The expert standard for oral health has been pilot-implemented in 18 stationary facilities in Germany. Nine residential long-term care facilities and 9 hospitals with capacities ranging from 275 to 3000 beds were examined; further, 8 outpatient care services were included [6]. However, 5 facilities discontinued the implementation at an early stage, with reasons cited as challenges such as staffing shortages due to the COVID-19 pandemic, high staff turnover, and temporary staff absences [6].

Following a 6- to 8-week period of implementation of the interventions, a self-administered evaluation was conducted in each facility, in which patient medical record analyses revealed that assessments of oral health by nursing staff were infrequent, particularly in residential long-term care facilities (73%) and hospitals (79%), compared to outpatient care (94%) [6].

Preliminary audit findings of the implementation process highlight the need for further research to enable a comprehensive evaluation of the implementation process, to understand how the standard is received by nursing staff, and to identify factors that influence implementation [5].

The audit results also indicate that acceptance is generally rated as satisfactory; however, significant differences exist across various care settings [6]. While some facilities successfully completed the implementation, others had to discontinue it prematurely. This highlights the variability in acceptance and implementation, which is influenced by factors such as the type of facility, available resources, and the specific patient population [6]. Such differences in acceptance and implementation effectiveness suggest that further research is necessary to understand the reasons behind these disparities and to develop strategies for overcoming them.

### The Expert Standard for “Oral Health in Nursing”

This expert standard, “promoting oral health in nursing,” was created by an interprofessional team of experts, comprising professionals from nursing, dentistry, and self-help organizations, to enhance oral health, particularly for older adult care–dependent individuals. Based on a systematic literature research, the group formulated consensus-based and practical recommendations [5].

The expert standard is conceptualized as a multicomponent intervention, comprising a set of distinct recommended interventions to be anchored in nursing practice. Each intervention is associated with a specific domain of action defined by the standard and should be assessed for its acceptability and feasibility before implementation [6,15].

These recommended interventions are as follows:

1. Standardized oral care procedure: regular oral care constitutes a pivotal measure for the promotion of oral health. It encompasses cleaning rituals twice a day, including tooth brushing, the use of toothbrushes, interdental cleaning tools, and mouthwashes, as well as the cleaning of dentures. Nurses bear the responsibility of implementing or providing assistance with these measures, particularly for patients who are incapable of performing them independently. The objective of regular oral care is to prevent the accumulation of plaque and the development of caries, thereby reducing the risk of infections.
2. Systematic assessment of oral health: it is recommended that systematic screenings and assessments of oral health be conducted at the beginning of the care process and as needed. Regular evaluations ensure that oral health is continuously monitored and addressed according to the identified needs.
3. Training and continuing education: nursing professionals must receive comprehensive training to adequately perform oral hygiene tasks. Regular continuing education ensures that nursing staff apply up-to-date methods and knowledge. This supports professional and patient-centered care practices, which are essential for preventing oral health problems.
4. Interprofessional collaboration: close cooperation with dentists is recommended to ensure comprehensive and interdisciplinary care. Coordinating this collaboration is essential for providing holistic care and treatment.
5. Promoting oral health in specific conditions, such as dry mouth: while specific measures to address conditions such as dry mouth are not explicitly outlined, the recommendations include general measures to promote oral health. These also encompass the treatment and management of specific issues such as dry mouth.
6. Establishment of an oral hygiene manager: although not explicitly included in the expert standard, Dr Elmar Ludwig, a co-author of the expert standard, proposed the introduction of an oral hygiene manager during the interdisciplinary symposium “Day of Senior Dental Medicine” on January 27, 2024, in Düsseldorf, Germany (personal communication by Dr Elmar Ludwig, Committee for Gerodontology, German Dental Association, Berlin, Germany, on January 27, 2024). This role would serve as a central coordination point within care facilities, overseeing all aspects of oral care and ensuring that nursing staff are adequately trained and equipped. The oral hygiene manager would also coordinate collaboration with dentists and other professionals, ensuring consistent and high-quality implementation of oral health standards, ultimately improving care for those in need.

The current state of research reveals that, despite the evident importance of interprofessional approaches to promoting oral health in older patients within clinical settings, specific data on the effectiveness and acceptance of such programs remains scarce [19]. The concept of the expert standard for oral health, introduced in Germany, was primarily developed through consensus decisions rather than empirical data [5]. Although this standard has been implemented as a pilot in a variety of facilities, studies confirming its acceptability, feasibility, and effectiveness are still lacking [6].

### Theoretical Framework of Acceptability

**Figure 1:**
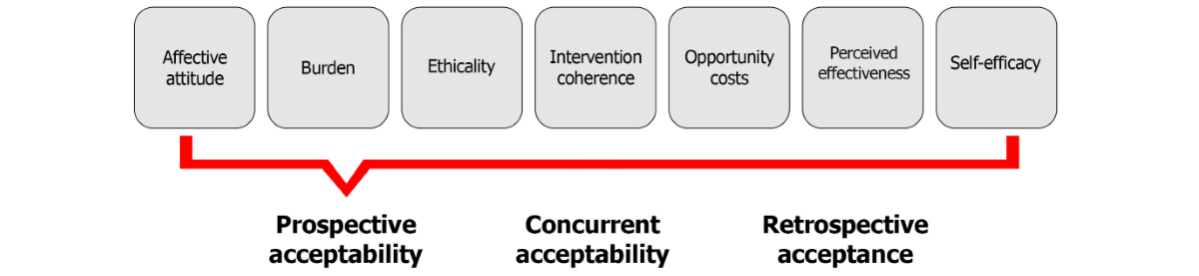
The TFA model with 7 key components [20]. TFA: theoretical framework of acceptability.

The theoretical framework of acceptability (TFA), developed by Sekhon et al [20], offers a multidimensional definition of acceptability. It assesses how individuals involved in delivering or receiving a health intervention perceive its suitability, considering their expected or actual emotional and cognitive responses. The TFA identifies 7 key components [20]: affective attitude (describing the emotional state toward the intervention), burden (measuring the perceived effort involved), intervention coherence (reflecting the level of understanding of the intervention), ethicality (examining the compatibility with personal values), opportunity costs (assessing necessary sacrifices), perceived effectiveness (estimating the expected success of the intervention), and self-efficacy (gauging confidence in one’s ability to perform the intervention; Figure 1).

Acceptability is potentially critical in settings where interprofessional approaches are required, as different professional groups often have varying perspectives and expectations of an intervention. This diversity can either facilitate or hinder implementation, depending on how well the intervention aligns with the needs and workflows of the different groups [5].

Moreover, it is essential to understand acceptability not as a 1-time act but as a continuous process [20]. The sustainability of an implementation depends on whether the intervention can be integrated into the daily routines of users and whether it continues to be perceived as useful and relevant.

### Nursing Competence as a Predictor for Acceptability of the Expert Standard

In this work, competence is understood as the ability to apply learned knowledge in a professional context, act in a self-organized manner, and make independent decisions [21]. According to Erpenbeck [22], competence is more than a mere qualification. It encompasses cognitive as well as action-oriented, social, and normative dimensions. While the cognitive dimension refers to knowledge and understanding, the action-oriented dimension focuses on the application of skills. The social dimension includes communication skills and responsibility, and the normative dimension describes the internalization of values, rules, and norms. These dimensions form the foundation for actions that are both professionally and morally sound, as well as self-organized [22].

**Figure 2:**
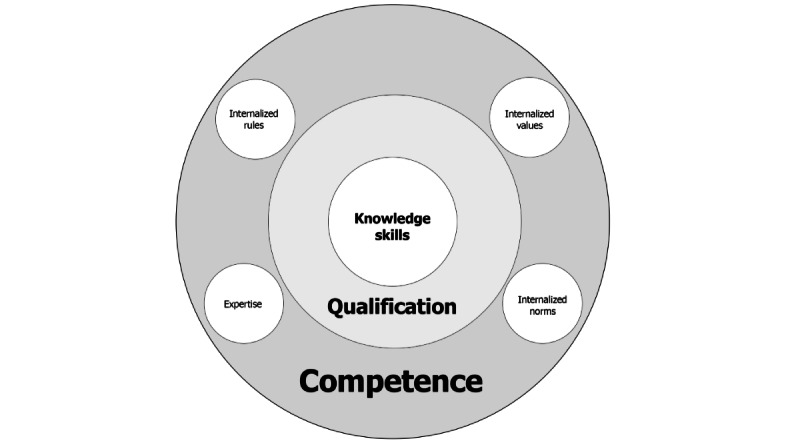
Inclusion model based on Erpenbeck et al [23].

The inclusion model proposed by Erpenbeck provides a framework for assessing and developing competencies (Figure 2 [23]).

The question arises as to whether a high level of nursing competence, specifically oral care competence, is a contributing factor to the greater acceptability of the expert standard intervention.

It can be argued that a higher level of competence positively influences the domains of the TFA. Competence development through practice and knowledge acquisition not only enhances self-efficacy [24] but is also expected to have a positive impact on coherence and perceived effectiveness [25]. Furthermore, the authors speculate that competent actions are likely to reduce the perceived burden.

**Figure 3:**
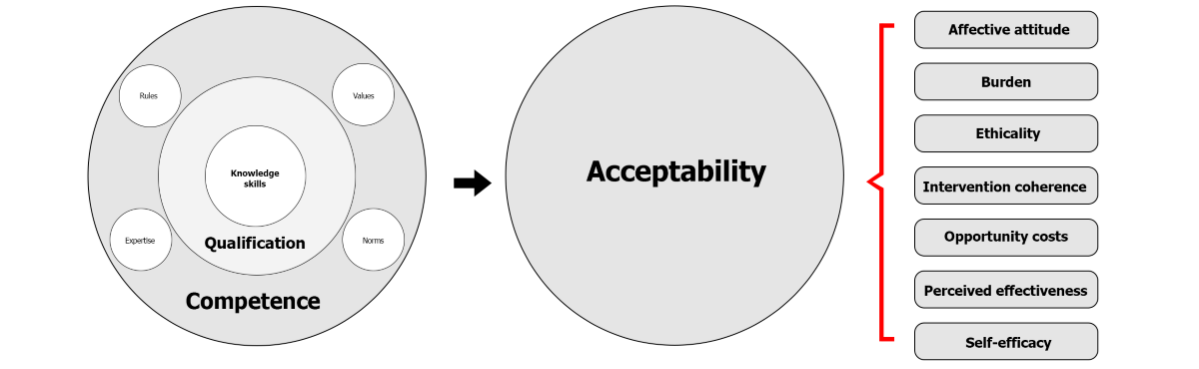
Combined acceptability model.

Therefore, the hypothesis is proposed that nursing competence, measured in terms of qualification level, oral health literacy, and oral care competence, has a positive effect on the acceptability of the intervention (Figure 3).

### Research Objectives and Questions

This study pursues 3 objectives: first, to determine the rate of acceptability of the several interventions derived from the expert standard. Second, to examine the influence of competence (knowledge, qualifications, and skills) on acceptability. Third, to assess the extent of CROM in oral health care and its relationship to the acceptability of new interventions.

The successful implementation of new measures requires broad acceptance among recipients. Currently, there is a lack of empirical data on this topic, which represents the first research gap. The authors assume a relationship between competence and acceptability. Several interdisciplinary theories describe potential influences of competence on the domains of the TFA.

Primary objective: estimate the acceptability rate and level for the interventions of the expert oral health care standard in various residential long-term care facilities and hospitals.

### Primary Research Questions

Question (Q; Q1): what is the acceptability (in terms of acceptability rate and level) of the individual interventions from the expert standard for promoting oral health among nursing staff in residential long-term care facilities and hospitals?

Q2: which specific interventions from the expert standard are particularly acceptable, and which are not?

Q3: are there differences in the acceptability rate and level between long-term care facilities and hospitals?

Secondary objective: assess the competence of nursing staff and determine the relationship between competence level and the acceptability level of the interventions.

### Secondary Research Questions

Q4: what influence does the competence level of nursing staff have on their acceptability of interventions?

Q5: what obstacles are perceived in the implementation of interventions to promote oral health in older adult care?

Q6: how important is oral care in the care of older individuals?

Q7: is there a relationship between the severity of subjectively perceived obstacles and the acceptability of interventions?

Tertiary objective: measure CROM in oral health care and identify the relationship between the CROM phenomenon and the acceptability level of interventions.

### Tertiary Research Questions

Q8: how do CROM rates in oral care in Germany differ from already published CROM rates in Austria [13] and Switzerland [11]?

Q9: is there a relationship between the nursing staff’s acceptability of new interventions and the CROM rate in oral care?

### Hypotheses

The formulation of the hypotheses is based on prior observations and theoretical considerations suggesting that variations in the implementation of standards and the competence of nursing staff are critical factors influencing the acceptability of health interventions. Additionally, it is assumed that both structural and individual differences within facilities can have a significant impact on the outcomes. By clearly defining these assumptions, the hypotheses can be systematically tested, highlighting the practical relevance of this study’s results and their implications for further research.

### Hypotheses for the Primary Research Questions

Without hypothesis: the estimation of the acceptability rate and level of the interventions from the expert standard will be conducted without additional hypothesis formulation.

Hypothesis (H; H1): the interventions from the expert standard differ in terms of their acceptability level.

H2: the acceptability level of the interventions varies between the types of facility (different residential long-term care facilities and hospitals).

**Hypotheses for the Secondary Research Questions**

H3: there is an association between the competence of nursing staff in oral care and the acceptability level of interventions from the expert standard.

H4: there are differences in the severity of obstacles perceived as barriers to oral care.

H5: there is an association between the subjectively perceived severity of obstacles to performing oral care measures and the acceptability level of interventions from the expert standard.

**Hypotheses for the Tertiary Research Questions**

H6: there is an association between CROM in oral care and the acceptability of interventions from the expert standard.

H7: there are differences in the CROM rate for oral care between Germany and already published CROM rates in Austria [13] and Switzerland [11].

## Methods

### Study Design

This is a quantitative cross-sectional study in which data are collected from nursing staff in hospitals and long-term care facilities at a specific time using standardized survey instruments. This study protocol was created based on SPIRIT (Standard Protocol Items: Recommendations for Interventional Trials) 2013 Guidelines [26]. According to the MRC Framework for Developing and Evaluating Complex Health Interventions, this research is part of the feasibility evaluation [15]. The first phase—identifying or developing the interventions of the expert standard—has already been completed [5]. The third and fourth phases—implementation and evaluation—are the focus of this research and have yet to be conducted.

### Setting and Study Population

This study is conducted in North Rhine-Westphalia, Germany’s most populated federal state, with 18.2 million residents [27]. It includes various care facilities, such as hospitals and university clinics with geriatric wards, as well as long-term care facilities.

This study population consists of nursing staff working in the mentioned facilities. The following inclusion criteria are applied: nursing staff who perform or oversee daily oral care for older adults. Exclusion criteria are trainee or voluntary staff.

### Outcome Measures and Data Collection

#### Acceptability

The TFA questionnaire is an assessment tool developed to measure the acceptability of health interventions. It consists of 7 items, each representing 1 dimension of intervention acceptability: affective attitude, burden, intervention coherence, perceived effectiveness, self-efficacy, ethical acceptability, and opportunity costs. Additionally, the questionnaire includes 1 item assessing the overall acceptability of the specific expert standard intervention [20,28].

Each item on the TFA questionnaire is typically rated on a 5-point rating scale, allowing quantitative measurement of agreement or disagreement with each statement. The questionnaire is specifically designed to capture the multidimensional aspects of acceptability, which is crucial for understanding how and why certain interventions are accepted or rejected by health care professionals [20,28].

For our study, the TFA questionnaire was specifically adapted to evaluate the acceptability of each intervention within the expert standard for promoting oral health in nursing care. Each specific intervention—including regular oral care, assessment and evaluation of oral health, training and continuing education for nursing staff, collaboration with dental professionals, and specialized measures to address dry mouth—is assessed through the 7 specific items in the TFA questionnaire. This adaptation results in a total of 35 items. The items were formulated according to the guidelines suggested by Sekhon et al [28].

The TFA questionnaire can be applied to assess both prospective acceptability (before implementation) and retrospective acceptance (after implementation), evaluating how interventions are perceived before and after their introduction [20,28]. Our planned study will investigate the prospective acceptability of these measures. This means that the survey will be conducted in institutions where the interventions from the expert standard have not yet been introduced.

For each specific intervention of the expert standard, a descriptive text was created detailing the content, core elements, and specific tasks. This was included to provide nursing staff with clear guidance and support in understanding the content of the specific expert standard.

Furthermore, it was decided to use a 6-point rating scale instead of a 5-point scale as the response format. The rationale for this decision is explained in the section on adjustment of rating scales.

#### Nursing Competence

According to Erpenbeck [22], qualification involves the interplay of knowledge, skills, and abilities [22]. Building on Erpenbeck model, oral health knowledge is assessed using the Oral Health Literacy Profile (OHLP), nursing qualification is gathered through a sociodemographic survey, and nursing competence in oral care is measured using the questionnaire from the DNQP (Deutsches Netzwerk für Qualitätsentwicklung in der Pflege [German network for Quality Development in Nursing]). The data collection instruments used are described as follows.

#### About OHLP

The OHLP is a tool developed to assess an individual’s knowledge and behavior related to oral health. For our study, the Oral Health Knowledge module consisting of 10 questions was used [29]. These questions aim to evaluate respondents’ knowledge of various aspects of oral health, including the impact of diet on dental health, the importance of fluoride, general oral hygiene practices, the connection between oral health and systemic diseases.

Each question is presented in a single-choice or multiple-choice format.

Participants receive 1 point for each correct answer. Incorrect answers or the option “I don’t know” are not scored. Scores from the questions are combined into an overall score, normalized on a scale from 0 to 100, with higher values indicating better oral health knowledge [29].

#### Nursing Competence in Oral Health

The audit tool: questionnaire 2 (DNQP-FB2 [Deutsches Netzwerk für Qualitätsentwicklung in der Pflege Fragebogen 2]) from the DNQP includes a specialized questionnaire designed to assess the knowledge level and further educational needs in mouth care practices [6].

The questionnaire comprises 5 items, each rated on a 5-point rating scale ranging from “very good” to “poor.” Additionally, respondents are asked whether further training is needed for each topic, with a simple “yes” or “no” response option [6].

The items cover a wide range of areas: assessing risks and problems related to oral health, planning and coordinating interventions, providing information, training, and counseling, performing nursing interventions, and evaluating the effectiveness of these interventions. This structured approach helps identify specific knowledge gaps and facilitates targeted training to enhance the quality of oral care in nursing facilities [6].

The response scale for this questionnaire was also adapted to a 6-point rating scale to provide more nuanced feedback.

#### Subjectively Perceived Importance of Oral Care Measures

It is assumed that the evaluation of oral care measures as highly important can be defined as an expression of competence. This information is collected using a question adapted from the questionnaire by Gibney et al [30]. To determine the perceived importance of oral hygiene, respondents are asked to rate the importance of oral hygiene on a 5-point rating scale. The scale ranges from “not important” (anchored by “cleaning glasses”) to “extremely important” (anchored by “wound care”).

#### Barriers to Performing Oral Hygiene Measures

In the acute care nurses’ questionnaire on oral hygiene by Gibney et al [30], barriers to performing oral care are assessed by asking respondents to rank 8 potential obstacles from most to least significant [30]. To reduce the likelihood of invalid responses in a web-based survey, this ranking system was adapted. Instead of ranking, each of the 8 barriers is rated using a slider on a scale from 0 to 100, allowing participants to subjectively assess the severity of each barrier.

#### About CROM

To measure CROM, a question regarding oral and dental hygiene from the first module, “activities of daily living” in the Basel Extent of Rationing of Nursing Care Assessment Questionnaire [31], is used. The question is:

How often in the last 7 working days did it happen that you could not perform necessary oral or dental hygiene for a patient?

This question is typically answered on a 4-point scale (never, rarely, sometimes, or often). For consistency with the response format across the survey, the scale was adapted to a 6-point format. The revised scale ranges from “very rarely” to “very often.”

Both Zuniga et al [11], who used the Basel Extent of Rationing of Nursing Care Assessment Questionnaire [31], and Cartaxo et al [13], who used the MissCare Survey [32], dichotomized the responses to determine the proportion of nursing staff who rationed or omitted care. In the study by Zuniga et al [11], categories 3-4 were combined, while Cartaxo et al merged categories 4-6.

To ensure comparability with the national studies conducted in Austria [13] and Switzerland [11], this study includes the question with 2 timeframes: 7 days and 14 days, accommodating differences in data collection periods.

#### Sociodemographic Data

Sociodemographic data collected includes age, gender, years of practical experience, employment type, work shift, qualification level, and specific competency roles such as nursing aide, nurse, and nursing management. Additionally, data on facility size, unit type and size, setting (urban or rural), and legal status of the institution are gathered.

The age of nursing staff is considered a confounding factor in the acceptability of new interventions. Nurses of different ages often exhibit varying attitudes. Older nurses have been found to show lower acceptability toward the introduction of modern measures in nursing compared to younger colleagues [33]. However, older nurses often possess greater practical competence due to extensive professional experience [7].

The work shift (morning or evening) is also considered a factor influencing the acceptance and level of new nursing measures. Morning and evening shifts differ significantly in terms of workload and priority setting. While the morning shift involves a tightly packed schedule of nursing services and organizational and documentation tasks [34-36], the evening shift focuses on administering medication and serving meals [37,38]. These differences in workload could influence the perception of the usefulness and importance of new measures.

#### Exploratory Questions

Additional questions are included to determine whether the interventions from the expert standard are already known to the nursing staff and whether they are currently being applied.

### Translation and Adaptation of Questionnaires

The existing questionnaires by Sekhon et al [28] and Gibney et al [30] were translated by the authors into German and culturally adapted. As prescribed by Sekhon et al [28], the TFA was tailored to ensure that its wording and semantics correspond to the topic of the research. Experts in nursing science and oral health were consulted to ensure the appropriateness of the questions.

The TRAPD (Translation, Review, Adjudication, Pretesting, and Documentation) method was applied for the translation process. This team-based approach ensures the quality and cultural relevance of the translation through review, resolution of discrepancies, pretesting, and detailed documentation [39,40].

The translated and adapted questionnaires were initially reviewed in a small team consisting of a dentist, a statistician, a psychologist, and a nursing scientist. Using the Think Aloud Method [41], the questionnaires were tested for clarity and feasibility refined.

### Adjustment of Rating Scales to Optimize Data Quality

In our study, a modification was made to the originally used rating scales in the questionnaires by Sekhon et al [28], Gibney et al [30], and DNQP [6], replacing the 5-point scale with a 6-point scale. Each item is considered a Likert-type item, and the mean of such items represents a Likert scale [42].

This adjustment, based on the elaborations of Moosbrugger and Kelava [41], aims to enhance the scale’s ability to differentiate between responses and to reduce ambivalent answers by removing the neutral middle category. This increases the precision and validity of the data. Expanding the response options also improves the reliability of the measurement instruments and minimizes response biases by encouraging participants to take a clearer stance [41].

### Study Procedure

#### Selection of Hospitals

A total of 25 hospitals and long-term care facilities in North Rhine-Westphalia, Germany, will be selected. These are sites where the interventions of the expert standard have not yet been implemented.

#### Recruitment of Participants

The nursing management of the selected hospitals will be contacted to gain support for participant recruitment. Information sessions will be held to inform nursing staff about this study and encourage participation. Direct communication will be established with facility management or human resources departments to foster cooperation. Participation will be voluntary and anonymous.

#### Recruitment Communication Tools

A QR code linking to the web-based survey will be included with the nursing staff’s pay slips. Additionally, a flyer explaining the importance of this study and encouraging participation will accompany the pay slips. The QR code will be distributed repeatedly over a 3-month period, along with reminders to participate, until the desired number of participants is reached.

#### Data Collection

Data will be collected exclusively through an web-based survey. To minimize potential bias and ensure representativeness, the sample will reflect institutional diversity by surveying Germany’s most populous state. This approach ensures a representative range of institutions and captures ethnic diversity.

### Ethical Considerations

This study follows the ethical principles outlined in the Declaration of Helsinki and was reviewed and approved on January 14, 2025, by the Research Committee for Scientific Ethical Questions of the UMIT Tirol (Private University for Health Sciences and Health Technology) in Tirol, Austria (reference number 3512).

The selection of nursing staff, as well as the storage, transmission, and access to collected data, will be anonymized and accessible only to authorized personnel.

Participation in this study is entirely voluntary, and written informed consent will be obtained after participants have been fully informed about this study’s purpose, procedures, and any potential risks.

As an incentive, 30 gift cards valued at €20 (US $22.74) each will be raffled among participants. To enter the raffle, participants will be asked to provide their email addresses. In compliance with ethical committee requirements, participants’ contact information will be stored separately from their survey responses to maintain confidentiality and data protection.

### Statistical Analysis

#### Overview

This study protocol outlines the statistical considerations for this study. Categorical variables will be described using absolute frequencies and percentages. For variables measured on at least an interval scale, mean, median, SD, minimum, and maximum values will be calculated.

The assumptions for applying statistical methods will be examined in advance, and appropriate alternative methods will be used if assumptions are violated. In the case of the planned repeated measures ANOVA, specific attention will be given to testing sphericity for within-subject factors, homogeneity of variances for between-subject factors, and the absence of outliers. For regression analyses, the homoscedasticity of residuals and the absence of outliers will be assessed.

Identified confounders will be accounted for in the statistical models, with particular attention paid to multicollinearity among predictors.

The statistical analysis plan has been reviewed and approved by a certified statistician for its accuracy and suitability to address the research questions.

The data analysis will be performed using IBM SPSS Statistics (version 30.0; IBM Corp).

#### Primary Research Question: Acceptability Rate and Level

##### Calculation of Acceptability Rate and Level

The first objective of this study is to estimate the acceptability rate and level for each intervention within the expert standard. The items from the TFA questionnaire [28] serve as the basis for calculating these acceptability parameters.

The acceptability rate is determined by evaluating how many respondents selected a value of 4 or higher on a 6-point rating scale. Values 4-6 are considered indicators of higher acceptability, while values 1-3 represent lower acceptability. The rate is calculated by dichotomizing responses and determining the proportion of participants selecting the top 3 categories (4 to 6). A 95% CI will be generated for the acceptability rate. Each of the interventions of the standard will be analyzed separately.

For this analysis, frequencies (counts) and percentages (rates) will be reported separately for each TFA domain. Domains 2 (burden) and 7 (opportunity costs) will be reversed, as lower burden and lower opportunity costs indicate higher acceptability.

The acceptability rate for each intervention of the expert standard will be averaged across all 7 TFA domains. Similarly, for each TFA domain, the acceptability rate will be averaged across all 6 interventions, yielding a marginal distribution.

For every mean value calculated, a 95% CI will be provided.

##### Analysis of Differences in Acceptability Levels Across Domains and Interventions

A repeated measures ANOVA will be used to identify differences in acceptability levels between interventions and across the TFA domains. These calculations aim to test H1.

In a first step, the 6 interventions from the expert standard will be used as a within-subject factor to compare the average acceptability levels across the 7 TFA domains. Bonferroni-corrected post hoc analyses will be conducted to perform pairwise comparisons, identifying interventions with particularly high or low acceptability values.

In the second step, the model will be extended to include an additional within-subject factor representing the 7 TFA domains of acceptability. This will allow for analysis of differences in acceptability levels across the TFA domains and interactions of domains and interventions. This 2-step approach facilitates an in-depth examination of the acceptability structure.

##### Group Comparisons Regarding Acceptability Levels

The above-described repeated measures ANOVA model will be further expanded to include an additional independent variable to test H2. The variable “facility type” (hospital vs nursing home) will be added to the model to assess whether differences in acceptability levels are associated with the type of facility.

#### Secondary Research Questions: Competence and Barriers

##### Influence of Competence on Acceptability Level

**Figure 4:**
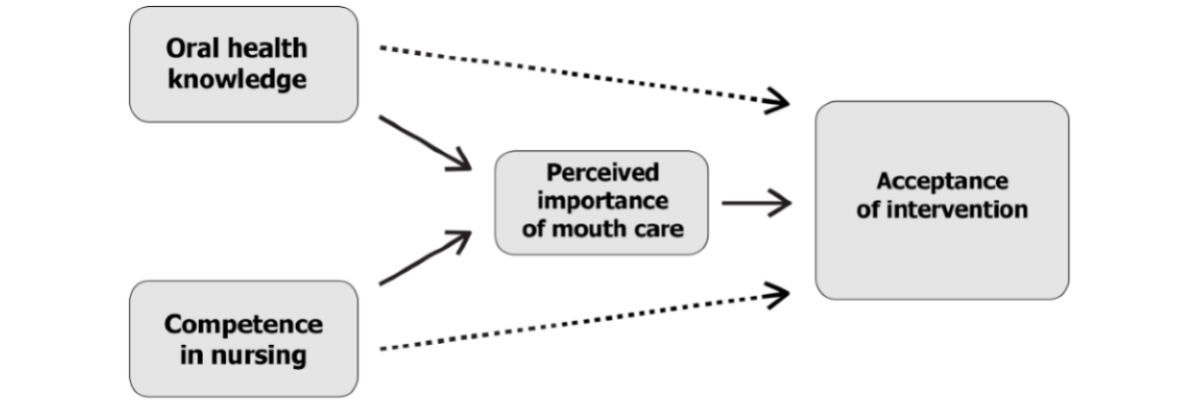
Regression analysis modelling acceptance using oral health knowledge and competence in nursing as predictors and perceived importance of mouth care as mediator.

To statistically analyze H3, a multiple linear regression analysis will be conducted (Figure 4). In our planned study, nursing competence in oral health will be operationalized using 2 measurement instruments, which will serve as predictors in the regression model. The first predictor is the sum score of the OHLP items, and the second is the mean index of the items from DNQP. Additionally, the subjectively perceived importance of oral health in the nursing process will be tested as a mediator to determine whether it mediates the relationship between knowledge or competence and acceptability.

The goal of these analyses is to examine the impact of these predictors on the acceptability level. First, an overall acceptability level will be calculated as the mean across all domains and interventions and used as the criterion in the regression model. Subsequently, the analysis will be repeated at the domain and intervention levels to calculate the impact of competence on each individual TFA domain and each intervention separately. This 2-step approach aims for a nuanced understanding of the influence of competence on acceptability.

##### Identification of Barriers to Implementing Mouth Care Interventions in Older Adult Care

The 8 identified barriers will be assessed using a rating scale. Absolute and relative frequencies for each barrier will be calculated, along with the mean and a 95% CI.

##### Influence of Barriers on Acceptability

A multiple linear regression analysis will be conducted, with the severity of the 8 identified barriers serving as predictors. Consistent with prior analyses, the overall acceptability level will first be calculated as a criterion and included in the regression. Subsequently, the analysis will be repeated at the domain and intervention levels to determine the influence of barrier severity on each TFA domain and each intervention separately.

##### Determining the Importance of Mouth Care

The subjectively perceived importance of mouth care will be analyzed by calculating absolute frequencies and percentages for each of the 5 levels on the rating scale. Additionally, the analysis will include a mean or median value to summarize the distribution of responses.

### Tertiary Research Questions: CROM

#### Estimation and International Comparison of the CROM Rate in Oral Care

The CROM rate for oral care will be calculated by assessing how many participants selected at least category 4 on a 6-point rating scale. Categories 4 to 6 will be used as indicators of rationed or omitted oral care, while categories 1 to 3 indicate no CROM. Through this dichotomization, the proportion of participants selecting the upper 3 categories will be calculated.

A 95% CI will be calculated for the CROM rate estimation.

To test H7, a chi-squared test of independence will be performed. The CROM rate for oral care identified in this study will be compared with previously published data from studies conducted in Austria [13] and Switzerland [11].

#### Exploratory Analyses

A multiple linear regression analysis will be conducted to examine the influence of sociodemographic characteristics on the acceptability level. The educational level of nursing staff will be included as a third predictor or mediator to explore whether educational qualification provides insight into knowledge, competence, and the perceived importance of oral health.

#### Significance Level for Hypothesis Testing

The significance level for all hypotheses is set at 5%. A Bonferroni correction will be applied when multiple statistical tests are conducted as part of hypothesis testing. Specifically, this is planned for post hoc analyses following repeated measures ANOVA and for the regression analysis that includes more than 1 predictor to assess the impact of nursing competence.

Due to the exploratory nature of this study, all results, particularly those related to interactions, will be treated as preliminary insights for this research area. These findings are intended to guide future studies with confirmatory designs for validation.

#### Sample Size Calculation

This study aims to estimate the acceptability rate of interventions from the expert standard with a precision of ±5 percentage points and a statistical confidence level of 95%. In the absence of specific assumptions about the acceptability proportion, a conservative estimate of 50% is used.

According to the BARMER Nursing Report [43], approximately 479,000 nursing staff were used in residential care facilities in Germany. However, the report does not provide a detailed breakdown of the proportions of skilled versus auxiliary staff [43].

Based on the large population size, the required sample size for estimating a proportion in the population is approximately 385 participants. The calculation for determining the sample size for population proportion estimation of nursing staff is as follows:




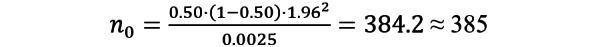




Additional calculations for statistical power will be performed to ensure that this study is sufficiently sensitive to detect the expected effects.

To address the second research question, a power analysis was conducted to evaluate the ability to detect a relationship between nursing competence and acceptability. Assuming a correlation coefficient (*r*) of 0.3 (explaining 9% of the variance), the power is calculated to be 99.9%, indicating a high likelihood of detecting such a correlation if it exists in the population.

Even with a smaller correlation of r=0.15 (explaining 2.3% of the variance), statistical significance is achievable, with a power of 84% to detect this correlation if present.

Thus, the sample size can be considered sufficient to address all research questions effectively.

#### Data Preparation

The data preparation process will be thoroughly documented. If response patterns in individual datasets suggest erroneous or intentionally inaccurate questionnaire completion, the respective participant will be excluded from the analysis, and the exclusion will be documented.

Such cases may include: clearly implausible responses (eg, a person indicates an age of 20 years while claiming 40 years of professional experience), inability to understand the questionnaire (eg, due to insufficient language proficiency), deliberately false answers (eg, intentional misinformation), uniform responses across all items, regardless of the content of the rating scale items.

All statistical analyses will be conducted following the available case analysis approach. Participants who do not complete the entire questionnaire will not be excluded from this study. Instead, only fully completed modules will be included in the corresponding analysis.

## Results

This study received approval from the Research Committee for Scientific Ethical Questions of the UMIT Tirol, Austria, in January 2025. Recruitment will commence in June 2025. Data collection and analysis are expected to be completed within 6 months. This study’s results will be submitted for publication in peer-reviewed scientific journals and presented at expert conferences. Additionally, it is planned to get in contact with decision makers.

## Discussion

### Principal Findings

This study aims to address a critical gap in the research surrounding the acceptance of the expert standard “promoting oral health in nursing” in German care facilities. By investigating the factors that influence the acceptance of this standard among nursing staff, this study provides essential insights into optimizing its implementation. The anticipated findings will highlight how the standard can be better integrated into routine practice, ultimately reducing the prevalence of CROM and improving the quality of oral health care for older populations. Furthermore, this research emphasizes the role of acceptability as a key determinant of feasibility, as interventions that are not acceptable are unlikely to be implemented effectively.

This study is expected to identify specific barriers and facilitators to the acceptability of the expert standard, such as nursing staff’s competence levels, workload, and the organizational support provided by care facilities. These findings will inform targeted strategies for enhancing the acceptability and feasibility of new health care standards, such as tailored training programs and interprofessional collaboration initiatives. Additionally, this research will contribute to a better understanding of how nursing staff perceive oral health interventions and the factors that impact their willingness to adopt new practices.

By focusing on acceptability as a prerequisite for successful implementation, this study also offers broader implications for the development of health care standards in nursing settings. The findings will support the creation of evidence-based policies that align with the realities of nursing care, addressing the unique challenges posed by high patient-to-nurse ratios and the increasing care needs of aging populations.

### Limitations

This study is limited by its reliance on self-reported data, which may be influenced by social desirability bias. Additionally, the cross-sectional design precludes an analysis of how acceptance changes over time or in response to specific interventions. Another limitation is that this study focuses on German care facilities, which may limit the generalizability of the findings to other health care systems or cultural contexts. Finally, resource constraints may impact the representativeness of the sample, as this study relies on voluntary participation.

Furthermore, the absence of qualitative methods is a limitation. While this study focuses on quantitative measurement, qualitative approaches could have revealed individual motivations and helped identify barriers to acceptability not captured by structured survey items.

However, qualitative approaches were used by the DNQP in the development of the expert standards. In the current phase of implementation according to the MRC framework, it is assumed that quantitative data collection will provide helpful and practice-relevant results. The quantitative method allows a large number of participants to be involved with the available resources. As the expert standard is to be widely implemented, the authors considered it important to achieve a large sample.

### Conclusion

This study will provide valuable insights into the factors influencing the acceptability of the expert standard for oral health in nursing care. By identifying barriers and facilitators, it aims to optimize the implementation of the standard, reduce the prevalence of CROM, and improve the quality of oral health care for older adult patients. The findings will not only contribute to the advancement of this specific standard but also offer important implications for the broader implementation of health care standards in nursing settings. Emphasizing the importance of acceptability and feasibility, this research underscores the need for evidence-based approaches to standard development and implementation, ultimately improving patient outcomes and supporting overburdened nursing staff in their critical roles.

## Abbreviations

CROM: care rationed or missed
DNQP: Deutsches Netzwerk für Qualitätsentwicklung in der Pflege (German network for Quality Development in Nursing)
DNQP-FB2: Deutsches Netzwerk für Qualitätsentwicklung in der Pflege Fragebogen 2
MRC: Medical Research Council
OHLP: Oral Health Literacy Profile
SPIRIT: Standard Protocol Items: Recommendations for Interventional Trials
TFA: theoretical framework of acceptability
TRAPD: Translation, Review, Adjudication, Pretesting, and Documentation
UMIT: Private University for Health Sciences and Health Technology

## Data Availability

The datasets generated during this study will not be publicly available until this study is completed and results are published. Upon reasonable request, data may be shared for scientific purposes. Requests can be directed to the corresponding author.
